# *PIK3CA* Mutations Frequently Coexist with *RAS* and *BRAF* Mutations in Patients with Advanced Cancers

**DOI:** 10.1371/journal.pone.0022769

**Published:** 2011-07-29

**Authors:** Filip Janku, J. Jack Lee, Apostolia M. Tsimberidou, David S. Hong, Aung Naing, Gerald S. Falchook, Siqing Fu, Rajyalakshmi Luthra, Ignacio Garrido-Laguna, Razelle Kurzrock

**Affiliations:** 1 Department of Investigational Cancer Therapeutics (Phase I Clinical Trials Program), The University of Texas MD Anderson Cancer Center, Houston, Texas, United States of America; 2 Department of Biostatistics, The University of Texas MD Anderson Cancer Center, Houston, Texas, United States of America; 3 Molecular Diagnostic Laboratory, The University of Texas MD Anderson Cancer Center, Houston, Texas, United States of America; Roswell Park Cancer Institute, United States of America

## Abstract

**Background:**

Oncogenic mutations of *PIK3CA*, *RAS* (*KRAS*, *NRAS*), and *BRAF* have been identified in various malignancies, and activate the PI3K/AKT/mTOR and RAS/RAF/MEK pathways, respectively. Both pathways are critical drivers of tumorigenesis.

**Methods:**

Tumor tissues from 504 patients with diverse cancers referred to the Clinical Center for Targeted Therapy at MD Anderson Cancer Center starting in October 2008 were analyzed for *PIK3CA*, *RAS* (*KRAS*, *NRAS*), and *BRAF* mutations using polymerase chain reaction-based DNA sequencing.

**Results:**

*PIK3CA* mutations were found in 54 (11%) of 504 patients tested; *KRAS* in 69 (19%) of 367; *NRAS* in 19 (8%) of 225; and *BRAF* in 31 (9%) of 361 patients. *PIK3CA* mutations were most frequent in squamous cervical (5/14, 36%), uterine (7/28, 25%), breast (6/29, 21%), and colorectal cancers (18/105, 17%); *KRAS* in pancreatic (5/9, 56%), colorectal (49/97, 51%), and uterine cancers (3/20, 15%); *NRAS* in melanoma (12/40, 30%), and uterine cancer (2/11, 18%); *BRAF* in melanoma (23/52, 44%), and colorectal cancer (5/88, 6%). Regardless of histology, *KRAS* mutations were found in 38% of patients with *PIK3CA* mutations compared to 16% of patients with wild-type (wt)*PIK3CA* (p = 0.001). In total, *RAS* (*KRAS*, *NRAS*) or *BRAF* mutations were found in 47% of patients with *PIK3CA* mutations vs. 24% of patients wt*PIK3CA* (p = 0.001). *PIK3CA* mutations were found in 28% of patients with *KRAS* mutations compared to 10% with wt*KRAS* (p = 0.001) and in 20% of patients with *RAS* (*KRAS*, *NRAS*) or *BRAF* mutations compared to 8% with wt*RAS* (*KRAS*, *NRAS*) or wt*BRAF* (p = 0.001).

**Conclusions:**

*PIK3CA*, *RAS* (*KRAS*, *NRAS*), and *BRAF* mutations are frequent in diverse tumors. In a wide variety of tumors, *PIK3CA* mutations coexist with *RAS* (*KRAS*, *NRAS*) and *BRAF* mutations.

## Introduction

Recently, major discoveries in the molecular biology of human cancers along with an increased understanding of oncogenic mutations and cell signaling pathways led to the successful application of new targeted therapies in several cancers.[1], [2], [3], [4] These include the use of KIT kinase inhibitors in *KIT*-mutant gastrointestinal stromal tumors (GIST), ABL kinase inhibitors in *BCR-ABL*-positive chronic myelogenous leukemia (CML), EGFR tyrosine kinase inhibitors in *EGFR*-mutant lung cancers, and BRAF inhibitors in *BRAF*-mutant melanomas.[2], [3], [4], [5] It appears plausible that the most common cancers have been difficult to treat, in part because they are heterogeneous, with each subset of patients having different molecular abnormalities. Identifying relevant molecular subtypes within heterogeneous cancers is crucial to future targeted therapeutic progress.[6], [7]

Key signals that are putatively activated in different tumor types are in the phosphatidylinositol 3-kinase (PI3K)/AKT/mTOR and RAS/RAF/MEK signaling pathways, which regulate cell proliferation and growth, apoptosis, autophagy, invasion, and migration.[8], [9] Activation is frequently mediated by mutations in the p110α subunit of *PI3K, PIK3CA*, with most mutations (>80%) occurring either in exon 9, which codes for the helical domain, or exon 20, which codes for the kinase domain.[8] Preclinical studies suggested that *PIK3CA* mutations could predict response to PI3K inhibitors, although concomitant mutations in *RAS* (*KRAS*, *NRAS*) or *BRAF* might mediate resistance.[10]

Although several preclinical studies suggest that aberrations in the PI3K/AKT/mTOR and the MAP kinase pathway may co-exist, only limited studies in patients have been undertaken, and have mostly concentrated on colorectal cancer.[8], [10], [11] We, therefore, investigated the *PIK3CA, RAS* (*KRAS* and *NRAS*) and *BRAF* mutation status of a large group of patients (N = 504) with advanced cancers referred to the Clinical Center for Targeted Therapy (CCTT) at The University of Texas MD Anderson Cancer Center (MD Anderson). We demonstrate that across tumor types, patients often concomitantly harbor *PIK3CA* mutations and *RAS*/*BRAF* mutations. These findings in the clinical setting have important implications for the design of clinical trials and treatments with PI3K/AKT/mTOR and BRAF or MEK inhibitors.

## Methods

### Patients

We investigated the *PIK3CA*, *RAS* (*KRAS*, *NRAS*), *BRAF* mutation status of patients with advanced tumors and available tissue referred to the MD Anderson CCTT (phase I clinic) starting in October 2008. The registration of patients in the database, pathology assessment, and mutation analysis were performed at MD Anderson. Eligible patients were those referred for phase I clinical trials of targeted therapeutic agents who had a sufficient amount of tumor tissue available for *PIK3CA* and, if possible, for other mutation analyses. The study was conducted under the umbrella of The IMPACT protocol, which was approved by The University of Texas MD Anderson Cancer Center Institutional Review Board I.

### Tissue samples and mutation analyses

*PIK3CA*, *RAS* (*KRAS*, *NRAS*), *BRAF* mutations were investigated in archival formalin-fixed, paraffin-embedded tissue blocks or material from fine needle aspiration biopsy obtained from diagnostic and/or therapeutic procedures. All histologies were centrally reviewed at MD Anderson. Mutation testing was performed in the Clinical Laboratory Improvement Amendment–certified Molecular Diagnostic Laboratory within the Division of Pathology and Laboratory Medicine at MD Anderson. DNA was extracted from microdissected paraffin-embedded tumor sections and analyzed using a polymerase chain reaction-based DNA sequencing method for *PIK3CA* mutations in codons [c]532–554 of exon 9 (helical domain) and c1011–1062 of exon 20 (kinase domain). This included the mutation hot spot region of the *PIK3CA* proto-oncogene denoted by Sanger sequencing, following amplification of 276 bp and 198 bp amplicons, respectively; utilizing primers designed by the MD Anderson Molecular Diagnostic Laboratory. Whenever possible, in addition to *PIK3CA*, mutation analysis was done for *KRAS* and *NRAS* c12, c13, and c61 mutations of exons 1–2; and *BRAF* codon 595–600 mutations of exon 15 by pyrosequencing as previously described.[12]

### Statistical analysis

Fisher's exact test was used to assess the association among categorical variables and mutation status. All tests were two-sided, and a P value less than 0.05 was considered statistically significant. All statistical analyses were carried out using SPSS 17 computer software (SPSS Chicago, IL).

## Results

### Patients

A total of 504 patients with diverse advanced cancers were analyzed for the presence of *PIK3CA* mutations. Of these 504 patients, 367 (73%) patients were also tested for *KRAS* mutations, 225 (45%) for the presence of *NRAS* mutations, and 361 (72%) for *BRAF* mutations. Two-hundred-and-ninety (58%) were women and 214 (42%) were men. The median age was 57 years (range, 13 to 91 years). One-hundred-and-five (21%) patients had colorectal cancers, 62 (12%) ovarian cancers, 55 (11%) melanomas, 34 (7%) squamous cell cancers of head and neck, 29 (6%) breast cancers, 28 (6%) uterine cancers, 26 (5%) sarcomas, 22 (4%) non-small cell lung cancers (NSCLC), 16 (3%) thyroid cancers, 15 (3%) non-squamous cell cancers of head and neck, 14 (3%) squamous cell cervical cancers, 12 (2%) adenocarcinomas of esophagus and stomach, 11 (2%) pancreatic cancers, 8 (2%) cervical adenocarcinomas, 8 (2%) renal cancers and 59 (11%) had other tumor types. Patient characteristics are listed in Table 1.

**Table 1:** Patient characteristics.

Characteristic	Patients	
	N	%
Sex		
Male	214	42
Female	290	58
Age (years)		
<50	142	28
50–70	298	59
>70	64	13
Ethnicity		
Caucasian	401	79
African American	45	9
Hispanic	29	6
Asian	29	6
Tumor type		
Colorectal	105	21
Ovarian	62	12
Melanoma	55	11
Head & neck: squamous	34	7
Head & neck: non-squamous	15	3
Breast	29	6
Uterine	28	6
Sarcomas	26	5
Cervix: squamous	14	3
Cervix: adenocarcinoma	8	2
Non-small cell lung	22	4
Small cell lung	2	<1
Thyroid	16	3
Esophagus and stomach: adenocarcinoma	12	2
Pancreatic	11	2
Renal	8	2
Neuroendocrine	7	1
Cholangiocarcinoma	5	1
Thymoma	5	1
Hepatocellular	5	1
Urothelial and bladder	5	1
Skin: non-melanoma	4	<1
Vulvar	3	<1
Adrenocortical	3	<1
Mesothelioma	3	<1
Carcinoma of unknown primary	3	<1
Anal: squamous	2	<1
Brain	2	<1
Other	10	2

### PIK3CA mutations

**Figure 1:**
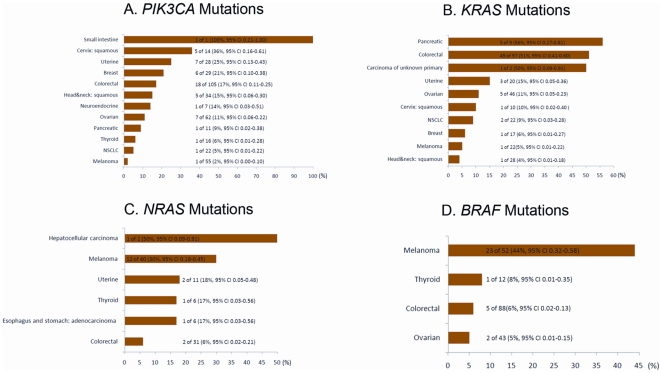
Frequency of mutations in tested tumors with 95% confidence intervals (CI). A. *PIK3CA* mutations. B. *KRAS* mutations. C. *NRAS* mutations. D. *BRAF* mutations.

*PIK3CA* proto-oncogene mutations were detected in 54 (11%) of the 504 patients. *PIK3CA* mutation status was not significantly associated with age, gender, or race. In tumor types with more than 10 patients tested, *PIK3CA* mutations were most common in squamous cell cervical cancer, in 5 (36%) of 14 patients. Mutations were also present in 7 (25%) of 28 patients with uterine cancer, 6 (21%) of 29 patients with breast cancer, 18 (17%) of 105 patients with colorectal cancer, 5 (15%) of 34 patients with squamous cell cancers of head and neck cancer, 7 (11%) of 62 patients with ovarian cancer, 1 (9%) of 11 patients with pancreatic cancer, 1 (6%) of 16 patients with thyroid cancer, 1 (5%) of 22 patients with NSCLC, and in 1 (2%) of 55 patients with melanoma (Figure 1A). Among disease entities with more than 10 patients tested, no *PIK3CA* mutations were found in sarcomas, and adenocarcinomas of stomach and esophagus.

**Figure 2:**
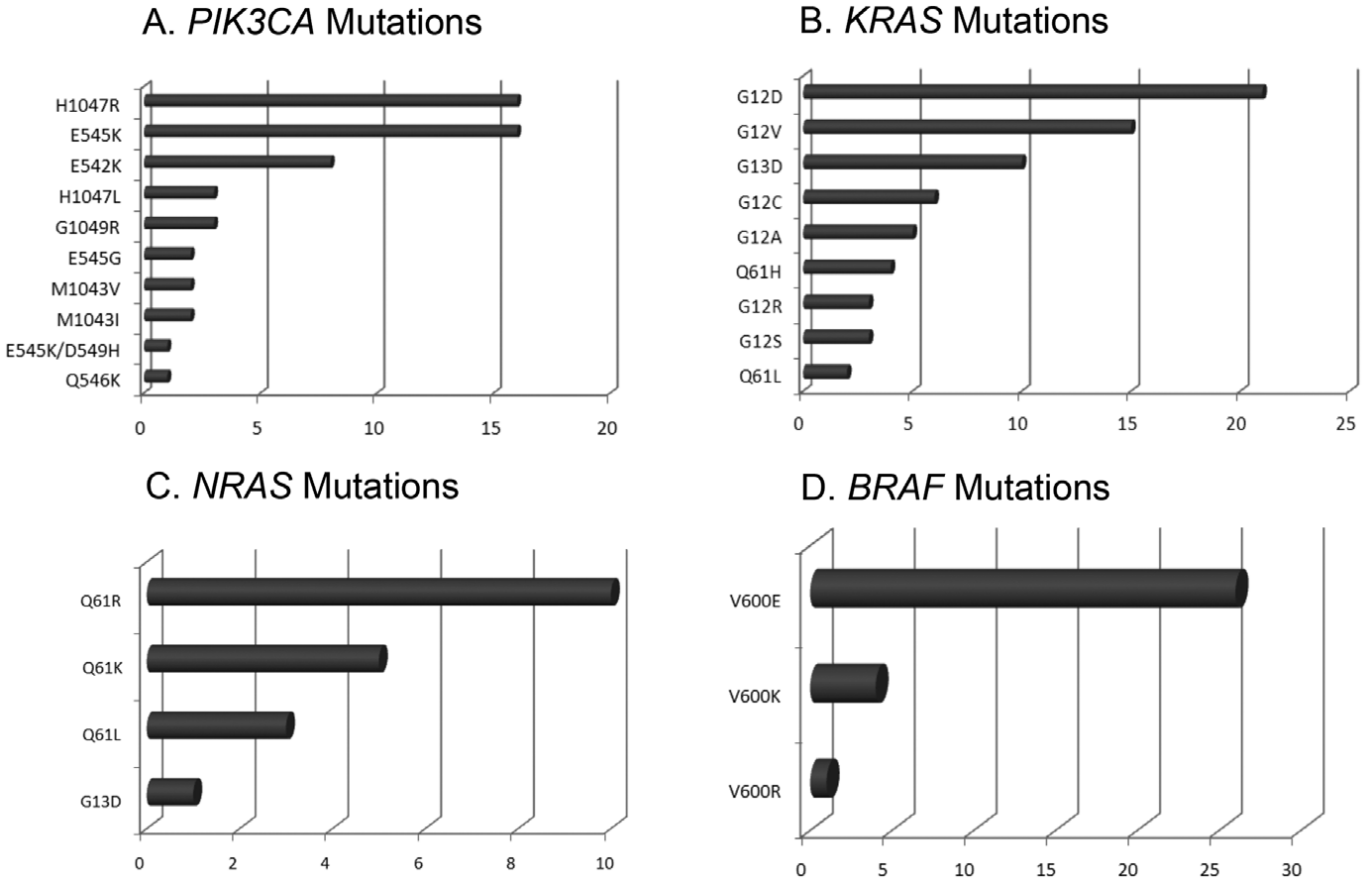
Proportion (numbers) of mutation types. A. *PIK3CA* mutations (n = 54). B. *KRAS* mutations (n = 69). C. *NRAS* mutations (n = 19). D. *BRAF* mutations (n = 31).

Mutations in exon 9 coding for the helical domain (E545K, E542K, E545G, E545K/D549H, Q546K) were found in 28 patients. Exon 20 mutations coding for the kinase domain (H1047R, H1047L, G1049R, M1043V, M1043I) were found in 26 patients. The most frequent mutations were H1047R (a mutation in codon 1047 of *PIK3CA* that changes the encoded amino acid from histidine to arginine) and E545K (a mutation in codon 545 of *PIK3CA* that changes the encoded amino acid from glutamic acid to lysine), each occurring in 16 (30%) of 54 patients with *PIK3CA* mutations (Figure 2A). In tumor types with at least 5 *PIK3CA* mutations identified, analysis of frequency of mutations in the helical vs. kinase domain was carried out. A predominance of helical domain *PIK3CA* mutations was observed in patients with cervical squamous (100% vs. 0%), colorectal (67% vs. 33%), and squamous cell cancer of head and neck (60% vs. 40%), while *PIK3CA* kinase domain mutations were predominant in patients with uterine (86% vs. 14%), breast (83% vs. 17%), and ovarian cancer (71% vs. 29%; p = 0.002).

We analyzed frequencies of *PIK3CA* mutations in different disease types in which specific mutations were identified in at least 5 tumor samples. In colorectal cancer, the most prevalent mutation was E545K (8/18, 44%); in uterine cancer, H1047R (4/7, 57%); in ovarian cancer, H1047R (3/7, 43%), in breast cancer H1047R (4/6, 67%), in cervical cancer E545K (4/5, 80%), and in squamous cell cancer of head and neck E542K (2/5, 40%). The small number of patients in each subgroup precluded performing a more detailed statistical analysis.

### KRAS mutations

*KRAS* proto-oncogene mutations were detected in 69 (19%) of 367 patients tested.

In tumor types with more than 10 patients tested, *KRAS* mutations were most frequent in colorectal cancer, in 49 (51%) of 97 tested patients. *KRAS* mutations were also present in 3 (15%) of 20 tested patients with uterine cancer, in 5 (11%) of 46 tested patients with ovarian cancer, 2 (9%) of 22 assessed patients with NSCLC, 1 (6%) of 17 tested patients with breast cancer, 1 (5%) of 22 tested patients with melanoma, and in 1 (4%) of 28 tested patients with squamous cell cancer of head and neck (Figure 1B). Among disease entities with more than 10 patients tested, no *KRAS* mutations were found in sarcomas, and adenocarcinomas of stomach and esophagus. *KRAS* mutation status was not significantly associated with age, gender, or race.

Mutations in c12 were found in 53 patients, c13 mutations in 10 patients, and c61 mutations in 6 patients. The most frequent mutation was G12D (a mutation in codon 12 of *KRAS* that changes the encoded amino acid from glycine to asparagine) detected in 21 patients (Figure 2B). We analyzed the frequencies of specific *KRAS* mutations in different disease types, with mutations identified in at least 5 tumor samples. In colorectal cancer, the most prevalent mutation was G12D (15/49, 31%); in ovarian cancer Q61H (2/5, 40%), and in pancreatic cancer G12V (2/5, 40%) and G12R (2/5, 40%). The small patient numbers in each subgroup precluded performing a more definitive statistical analysis.

### NRAS mutations

*NRAS* proto-oncogene mutations were found in 19 (8%) of 225 patients analyzed. In tumor types with more than 10 patients tested, *NRAS* mutations were most frequent in melanoma, in 12 (30%) of 40 tested patients. Mutations were also present in 2 (18%) of 11 tested patients with uterine cancer and in 2 (6%) of 31 tested patients with colorectal cancer (Figure 1C). Among disease entities with more than 10 patients tested, no *NRAS* mutations were found in ovarian cancer, and squamous cell carcinoma of head and neck. *NRAS* mutation status was not significantly associated with age, gender, or race.

Mutations in c61 were found in 18 patients, and 1 patient had a c13 mutation. The most frequent mutation was Q61R (a mutation in codon 61 of *NRAS* that changes the encoded amino acid from glutamine to arginine) detected in 10 patients (Figure 2C).

### BRAF mutations

*BRAF* proto-oncogene mutations were detected in 31 (9%) of 361 patients tested. In tumor types with more than 10 patients tested, *BRAF* mutations were most frequent in melanoma, in 23 (44%) of 52 tested patients. Mutations were also present in 1 (8%) of 12 tested patients with thyroid cancer, in 5 (6%) of 88 tested patients with colorectal cancer, and in 2 (5%) of 43 tested patients with ovarian cancer (Figure 1D). Among disease entities with more than 10 patients tested, no *BRAF* mutations were found in squamous cell cancers of head and neck, uterine cancers, breast cancers, NSCLC, sarcomas, and adenocarcinomas of stomach and esophagus. *BRAF* mutation status was not significantly associated with age, gender, or race.

All mutations were in c600. The most frequent mutation was V600E (a mutation in codon 600 of *BRAF* that changes the encoding amino acid from valine to glutamic acid) in 26 patients (Figure 2D).

### Simultaneous PIK3CA and RAS (KRAS, NRAS) or BRAF mutations

**Figure 3:**
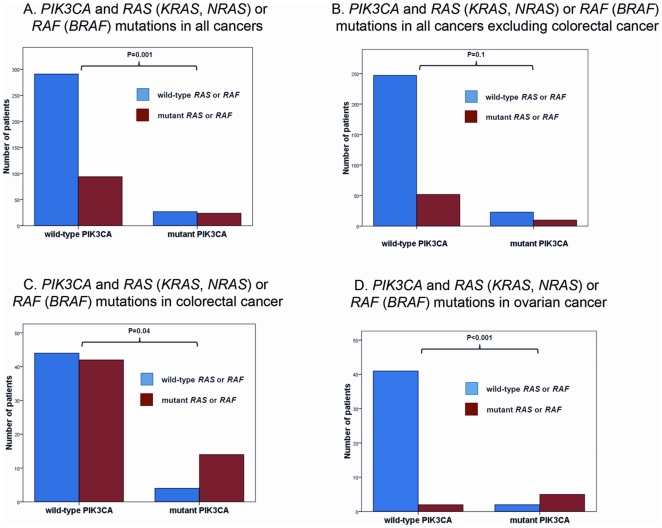
Simultaneous *PIK3CA* and *RAS* (*KRAS, NRAS*) or *BRAF* mutations. Wild-type *RAS* (*KRAS, NRAS*) or *BRAF* (blue bar) and mutant *RAS* (*KRAS, NRAS*) or *BRAF* (red bar) in: A. All tumor types (tested, n = 436); B. All cancers excluding colorectal cancers (tested, n = 332); C. Colorectal cancers (tested, n = 104); D. Ovarian cancers (tested, n = 50).

Either *RAS* (*KRAS*, *NRAS*) or *BRAF* mutations were more common in patients with mutant *PIK3CA* than in those with wild-type (wt) *PIK3CA* (p = 0.001) (Figure 3A). These mutations were found in 24 (47%) of 51 patients with mutant *PIK3CA*, who were also tested for *RAS* (*KRAS*, *NRAS*) or *BRAF* mutations, but only in 94 (24%) of 385 patients with wt*PIK3CA*, who were also tested for *RAS* (*KRAS*, *NRAS*) or *BRAF* mutations (Table 2). Similar associations between the proportion of *RAS* (*KRAS*, *NRAS*) or *BRAF* mutations in mutant *PIK3CA* and *wtPIK3CA*, although not always statistically significant, were found in disease-specific subanalysis in colorectal cancer (14/18 [78%] vs. 42/86 [49%]; p = 0.04), ovarian cancer (5/7 [71%] vs. 2/43 [5%]; p<0.001), and all tested cancers excluding colorectal (10/33 [30%] vs. 52/299 [17%]; p = 0.1) (Figure 3B–D).We also analyzed the frequency of *PIK3CA* mutations in patients with mutant *RAS* (*KRAS*, *NRAS*) or *BRAF* compared to patients without *RAS* (*KRAS*, *NRAS*) or *BRAF* mutations. Patients with *RAS* (*KRAS*, *NRAS*) or *BRAF* mutations had a higher frequency of *PIK3CA* mutations (24 of 118 patients, 20%) compared to those without *RAS* (*KRAS*, *NRAS*) or *BRAF* mutations (27 of 318 patients, 8%; p = 0.001).

**Table 2:** PIK3CA, RAS (KRAS, NRAS), and BRAF mutations.

Oncogene	Mutated (%)	Total tested
*PIK3CA*	54 (11)	504
*KRAS*	69 (19)	367
*NRAS*	19 (8)	225
*BRAF*	31 (9)	361
*KRAS* in mutated *PIK3CA*	19 (38)	50
*KRAS* in wild-type *PIK3CA*	50 (16)	317
*RAS*/*BRAF* in mutated *PIK3CA*	24 (47)	51
*RAS*/*BRAF* in wild-type *PIK3CA*	94 (24)	385

**Figure 4:**
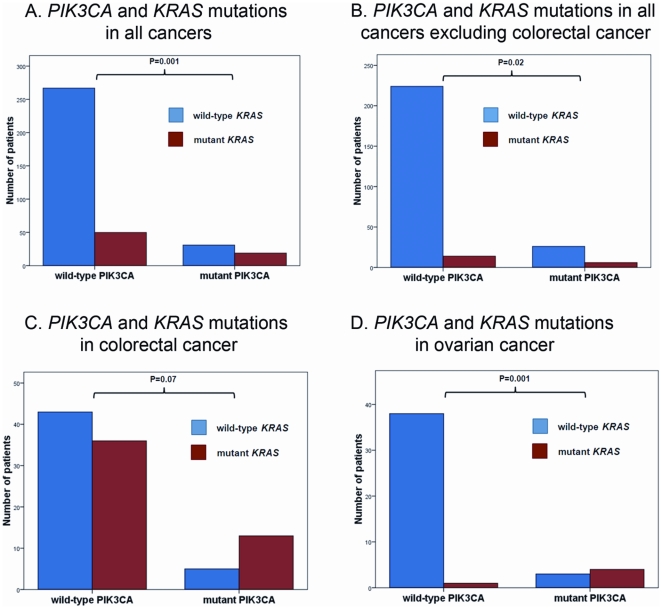
Simultaneous *PIK3CA* and *KRAS* mutations. Wild-type *KRAS* (blue bar) and mutant *KRAS* (red bar) in: A. All tumor types (tested, n = 367); B. All cancers excluding colorectal cancers (tested, n = 270); C. Colorectal cancers (tested, n = 97); D. Ovarian cancers (tested, n = 46).

When analyzing *KRAS* alone, these mutations were detected in 19 (38%) of 50 patients with *PIK3CA* mutations, who were also tested for *KRAS*. In patients with *wtPIK3CA*, *KRAS* mutations were found in 50 (16%) of 317 patients tested for both oncogenes (Table 2). The difference was statistically significant (p = 0.001) (Figure 4A). Similar associations between the proportion of *KRAS* mutations in patients with mutant *PIK3CA* and wt*PIK3CA*, although not always statistically significant perhaps because of smaller numbers of patients, were found in disease-specific subanalysis in colorectal cancer (13/18 [72%] vs. 36/79 [46%]; p = 0.07), ovarian cancer (4/7 [57%] vs. 1/39 [3%]; p = 0.001), and all tested cancers excluding colorectal (6/32 [19%] vs. 14/238 [6%]; p = 0.02) (Figure 4B–D). We also analyzed the frequency of *PIK3CA* mutations in patients with mutant *KRAS* vs. patients *wtKRAS*. Patients with *KRAS* mutations had a higher frequency of *PIK3CA* mutations compared to those *wtKRAS* (19/69 [28%] vs. 31/298 [10%]; p = 0.001). Finally, we analyzed associations between exon 9 *PIK3CA* and *KRAS* mutations and between exon 20 *PIK3CA* and *KRAS* mutations. Exon 9 *PIK3CA* mutations were strongly associated with *KRAS* mutations (12/62 [19%] in *KRAS* mutant vs. 16/283 [6%] in wt*KRAS*; p = 0.001), whereas the association between exon 20 *PIK3CA* and *KRAS* mutations did not reach statistical significance (7/57 [12%] in *KRAS* mutant vs. 15/282 [5%] in wt*KRAS*; p = 0.07). In addition, we analyzed associations between exon 9 *PIK3CA* and *KRAS* mutations and between exon 20 *PIK3CA* and *KRAS* mutations in colorectal and ovarian cancers, which were the two largest disease subgroups. In colorectal cancer, exon 9 *PIK3CA* mutations showed a trend towards increased frequency in patients with *KRAS* mutations (9/45; 20%) compared to patients with wt*KRAS* (3/46; 6%) (p = 0.07), whereas the frequency of exon 20 *PIK3CA* mutations did not significantly differ (4/40 [10%] in *KRAS* mutant vs. 2/45 [4%] in wt*KRAS*; p = 0.4). In ovarian cancer, there was a strong association between exon 20 *PIK3CA* and *KRAS* mutations (3/4 [75%] in *KRAS* mutant vs. 2/40 [5%] in wt*KRAS*; p = 0.003), while the association between exon 9 *PIK3CA* and *KRAS* mutations did not reach statistical significance (1/2 [50%] in *KRAS* mutant vs. 1/39 [3%] in wt*KRAS*; p = 0.1). However, numbers of patients in these subgroups were small, suggesting caution in interpreting these results.

There was no significant difference in the prevalence of *NRAS* mutations between *wtPIK3CA* and mutant *PIK3CA* groups, however, the small number of patients tested in the mutant *PIK3CA* group precluded drawing definite conclusions.

The proportion of *BRAF* mutations was similar (8–9%) in both *wtPIK3CA* and mutant *PIK3CA* groups. Low patient numbers in the mutant *PIK3CA* group made it problematic to arrive at definitive conclusions.

## Discussion

Across tumor types, we demonstrated a higher prevalence of *RAS* (*KRAS*, *NRAS*) or *BRAF* mutations (47%) and *KRAS* mutations (38%) in patients with mutant *PIK3CA* compared to those with *wtPIK3CA* (mutant *RAS* or *BRAF* present in 24%, p = 0.001; mutant *KRAS* present in 16%, p = 0.001). Most previously published studies investigating simultaneous *PIK3CA*, and *RAS* or *BRAF* mutations concentrated on colorectal cancer.[11], [13], [14], [15], [16] Some studies suggested that *PIK3CA* mutations are associated with *KRAS* mutations,[11], [13], [15] whereas others did not report that.[16], [17] A large retrospective study that analyzed 1,022 tumor DNA samples from patients with colorectal cancer treated with cetuximab in multiple European institutions revealed association between exon 9 *PIK3CA* and *KRAS* mutations (14.7% in *KRAS* mutant vs. 6.8% in wt*KRAS*; p = 0.0006), but not between exon 20 *PIK3CA* and *KRAS* mutations (3.8% in *KRAS* mutant vs. 2.3% in wt*KRAS*; p = 0.27).[11] In agreement with this paper, when we examined all histologies, we also found a strong association between exon 9 *PIK3CA* and *KRAS* mutations (19% in *KRAS* mutant vs. 6% in wt*KRAS*; p = 0.001), however, the frequency of exon 20 *PIK3CA* mutations also showed a trend towards being more common in patients with *KRAS* mutations compared to wt*KRAS* (12% in *KRAS* mutant vs. 5% in wt*KRAS*), albeit not reaching statistical significance (p = 0.07). In a disease-specific subanalysis in colorectal and ovarian cancer we noticed a trend toward an association between exon 9 *PIK3CA* and *KRAS* mutations in colorectal cancer (20% in *KRAS* mutant vs. 6% in wt*KRAS*; p = 0.07) and a statistically significant association between exon 20 *PIK3CA* and *KRAS* mutations in ovarian cancer (75% in *KRAS* mutant vs. 5% in wt*KRAS*; p = 0.003). However, the numbers of patients are low in colorectal, and ovarian cancer subgroup analyses suggesting that additional confirmatory studies will be necessary.

An association between *PIK3CA* and *RAS* or *BRAF* mutations has implications for cancer therapy. Preclinical models suggested that cell line-derived xenografts with *PIK3CA* mutations are sensitive to the PI3K inhibitor PX-866 unless they have *RAS* mutations.[10] Nearly identical findings were reported from preclinical and early clinical experiments with the mTOR inhibitor everolimus.[18] Similar observations have been reported from early clinical experiments when *RAS* or *BRAF* mutations in patients with mutant *PIK3CA* were associated with resistance to PI3K/AKT/mTOR in several cancers except for ovarian cancer.[19], [20] These data suggest that *PIK3CA* mutations might predict a response to PI3K/AKT/mTOR pathway inhibitors in only a portion of patients. In patients with simultaneous *PIK3CA* and *RAS* or *BRAF* mutations, PI3K/AKT/mTOR inhibition might not be sufficient for achieving a significant antitumor effect, and since *RAS* or *BRAF* mutations are common in patients with mutant *PIK3CA* it is advisable to determine the mutational status of *RAS* and *BRAF* in addition to *PIK3CA* status. Of special interest, clinical trials combining MEK and PI3K/AKT/mTOR inhibitors are in an early stage of clinical development.[21] In addition, some preclinical experiments suggested that PI3K inhibition might reduce the migration and adhesion of tumor cells and consequently inhibit metastasis rather than the primary tumor, which may have important implications for treatment; however, these observations need to be confirmed in additional experiments.[22], [23]

In regard to individual aberrations, oncogenic mutations in two hot spot regions (exons 9 and 20) of *PIK3CA* have been identified in various malignancies, including common tumors such as breast, lung, colorectal, ovarian and uterine.[11], [24], [25], [26], [27], [28], [29] In this study, *PIK3CA* mutations were identified in 11% of diverse tumor types. Tumors with a high prevalence of *PIK3CA* mutations were squamous cell cervical (36%), uterine (25%), breast (21%), colorectal (17%), squamous cell head and neck (15%), and ovarian cancers (11%). These data are similar to those previously published except for cervical cancer, which was shown to have a prevalence of *PIK3CA* mutations ranging from 8% (8/98) in the COSMIC database to 16% (2/12) published by Miyake et al.[29], [30] Colorectal and squamous cervical cancers were found to have a predominant E545K (exon 9) mutation (44%, 80%, respectively); uterine, ovarian, and breast cancers, a predominant H1047R (exon 20) mutation (57%, 43%, 67%, respectively); and squamous cell cancers of head and neck, an E542K (exon 9) mutation (40%). These differences may have clinical significance as some preclinical data generated a hypothesis that when exon 20 mutations are present in the kinase domain, they might be more sensitive to PI3K/AKT/mTOR inhibitors than exon 9 mutations in the helical domain.[18]

*KRAS* mutations occur in different tumor types, and are particularly important in colorectal, pancreatic, and NSCLC carcinogenesis.[11], [31], [32], [33], [34] *KRAS* mutations predict a lack of therapeutic benefit of anti-EGFR monoclonal antibodies in colorectal but, not convincingly, in lung cancer.[35], [36], [37] Functional RAS may also be potentially important for regulating the actin cytoskeleton, which was suggested as being a critical driver of oncogenic transformation.[38] In our study, a high prevalence of *KRAS* mutations was found in pancreatic (56%), colorectal (51%), uterine (15%), and ovarian (11%) cancers, which is similar to previously published findings and data from the COSMIC database.[11], [29] Colorectal cancers were found to have a predominant G12D mutation (31%); pancreatic cancers, G12V and G12R mutations (40% each); and ovarian cancers, Q61H mutations (40%). These distinctions may be clinically important as some preclinical data suggest that different mutations might activate different pathways. Ihle et al.[39] demonstrated that a G12D mutation activates both PI3K/AKT/mTOR and MAPK pathways, whereas a G12C mutation causes robust RAL signaling.

*NRAS* mutations have been mainly described in melanomas and leukemias and their prognostic significance has been unclear, with some data suggesting an association between mutant *NRAS* and a worse prognosis in melanoma.[40], [41], [42] In our study, there was a high prevalence of *NRAS* mutations in melanoma (44%), and uterine cancer (15%). The prevalence of *NRAS* mutations was higher than reported in other studies or in the COSMIC database (14–20%).[29], [43]

*BRAF* mutations have been mainly reported in melanoma, colorectal, papillary thyroid, and ovarian cancer.[44], [45] In colorectal cancer they are associated with a dismal prognosis, however, unlike *KRAS* mutations, *BRAF* mutations might not be predictive of lack of cetuximab benefit.[46] In melanoma, the prognostic significance of *BRAF* mutations is less obvious, although patients with *BRAF* mutant melanoma seem to respond very well to *BRAF* inhibitors.[4], [42], [47] In papillary thyroid cancer, *BRAF* mutations were found to upregulate microenviromental genes, which potentially increases tumor aggressiveness.[48] In agreement with previously published data, our study showed a high prevalence of *BRAF* mutations in melanoma (44%) and, to much lesser extent, in thyroid, colorectal, and ovarian cancer (8%, 6%, and 5%, respectively). Although the prevalence of *BRAF* mutations in thyroid cancer was much lower than the 51% previously published, this discrepancy could be explained by the presence of histologies other then papillary.[49]

In conclusion, we studied the prevalence of *PIK3CA*, *RAS* (*KRAS*, *NRAS*), and *BRAF* mutations in diverse tumor samples and identified a high frequency of coexisting *PIK3CA* and *BRAF* or *RAS* mutations. Simultaneous activation of PI3K/AKT/mTOR and RAS/RAF/MEK pathways can be associated with resistance to PI3K/AKT/mTOR inhibitors.[10], [18] These results are particularly important because of the many PI3K/AKT/mTOR and RAS/RAF/MEK targeting agents currently undergoing clinical testing and suggest that molecular profiling and matching patients with combinations of these targeted drugs will need to be investigated in depth.
